# Blockchain-Based Information Security Protection Mechanism for the Traceability of Intellectual Property Transactions

**DOI:** 10.3390/s25103064

**Published:** 2025-05-13

**Authors:** Zheng Wang, Wenlong Feng, Mengxing Huang, Siling Feng, Shilong Mo, Yunhong Li

**Affiliations:** School of Information and Communication Engineering, Hainan University, Haikou 570228, China; 23220854020010@hainanu.edu.cn (Z.W.); huangmx09@163.com (M.H.); fengsiling@hainanu.edu.cn (S.F.); 23220854020056@hainanu.edu.cn (S.M.); 23220854100028@hainanu.edu.cn (Y.L.)

**Keywords:** blockchain, intellectual property protection, PROV model, data traceability, threshold signature

## Abstract

Traditional intellectual property transaction traceability has problems such as information asymmetry, traceability information storage methods relying on centralized databases, and easy tampering of transaction information, etc. A blockchain-based information security mechanism for intellectual property transaction traceability is proposed. Firstly, through the analysis of massive intellectual property transaction case information, the commonality and individuality data are studied, and the structure and scope of data collection requirements for traceability information are established; secondly, the traceability information structure is constructed based on the smart contract and PROV data origin model, the signature verification of traceability information is completed based on the BLS threshold signature of the Dynamic DKG protocol, and the signature process integrates the PROV model and constructs a chained signature structure. The multi-level traceability information verification strategy and process are developed to achieve the security protection of traceability information throughout the entire life cycle of intellectual property transactions.

## 1. Introduction

In the era of the digital economy, intellectual property rights have become a core strategic resource within the national innovation system. According to the World Intellectual Property Organization (WIPO) Global Innovation Index Report 2023, China has been the world’s leading country in the creation of intellectual property rights (IPRs) such as patents and trademarks for six consecutive years, but the economic losses caused by IPR infringement have exceeded RMB 200 billion annually. This contradiction highlights the deep dilemma of the existing protection mechanism: although the traditional centralized registration system (e.g., the patent database of the State Intellectual Property Office) has legal authority, it is difficult to cope with the globalization and high-frequency demand for intellectual property transactions, and there are problems such as lagging traceability information and difficulties in cross-domain verification.

Blockchain technology provides a new solution path for intellectual property protection. Based on the characteristics of a decentralized ledger, existing research has realized the credible survival of intellectual property registration [1], transaction deposit [2], and other links. However, by analyzing blockchain intellectual property projects in recent years, it is found that current schemes generally have two key defects: first, blockchain-based traceability systems generally adopt simple hash uploading methods and lack structured descriptions of the origin of the data, which leads to difficulties in verifying the integrity of the information in judicial proof; second, most of the schemes rely on a single institution to sign and verify the signature, which creates a new centralized in cross-border transaction scenarios trust bottleneck. This directly affects the effectiveness of the judgment of the Court of Justice of the European Union (CJEU) in 2022 in the “blockchain copyright dispute case” and other typical cases—although the data on the chain has not been tampered with, due to insufficient verification of the data prior to uploading, the data was ultimately not admitted as valid evidence.

Article 11 of China’s Copyright Law provides that ownership of a work can be confirmed by means of a record of creation, proof of first publication, etc., and the Digital Rights Management (DRM) standard requires that digital content contain standardized metadata (e.g., creator, licensing terms, restrictions on use).

Aiming at the above problems, this paper proposes a dual verification mechanism that fuses data origin modeling and threshold signatures. Compared with the existing schemes, the innovative breakthrough of this study is reflected in three dimensions:

1. Enhanced legal compliance: The PROV data model is used to build an information traceability chain that complies with W3C standards, embedding blockchain timestamps to meet the legal requirements for the determination of the “time of first publication” and mapping the data of the entire life cycle of intellectual property rights creation, transaction, and use into judicially admissible elements of evidence.
2. Dynamic trust mechanism: design a BLS threshold signature scheme based on the dynamic DKG protocol, enabling multiparty collaborative verification by creators, trading platforms, regulatory agencies, etc., and the signature threshold can be dynamically adjusted with the trading scenario.
3. Chain verification structure: innovatively embedding the signature hash value of the previous stage into the subsequent verification link, forming an inseparable chain structure of traceable evidence, preventing man-in-the-middle attacks and timestamp forgeries.

## 2. Related Work

### 2.1. Blockchain Traceability Technology Evolution

The development of blockchain technology in the field of traceability has gone through three important stages: early simple transaction traceability represented by Bitcoin (2013–2016), mid-term smart contract-driven complex business traceability (2017–2020), and recent multi-technology fusion of intelligent traceability systems (2021–present). Through literature analysis, it is found that blockchain traceability research in the past three years presents three significant features:

1. The maturity of cross-chain technology: e.g., Ref. [3] adopts the super ledger Fabric multi-channel architecture to realize the data isolation and collaboration of different business sectors.
2. On-chain and off-chain collaborative storage: Ref. [4] reduces on-chain storage pressure while ensuring data trustworthiness through a hybrid storage scheme of IPFS and blockchain.
3. Cryptographic enhancement mechanism: Ref. [5] applies the state secret algorithm SM2 for identity authentication, and Ref. [6] combines blockchain smart contracts and other technologies to establish a drug tracking mechanism.

### 2.2. Technical Bottlenecks in Intellectual Property Traceability

Blockchain is one of the hottest emerging technologies today, and it has numerous innovations and developments in the field of traceability. Many researchers have proposed blockchain-based traceability solutions. Feng Guofu et al. [7] proposed a fish transaction traceability model based on the combination of blockchain and interstellar file system to ensure the reliable storage of transaction data in response to the problems of centralization, easy of tampering, data that cannot be shared, and credible traceability existing in the traditional aquatic products transaction traceability. P. Liu et al. [8] proposed to track the transactions of each node of the electric power supply chain based on the blockchain smart contract and hash algorithm, which realizes safe and reliable traceability of the power supply chain, enhances the commercial value of traceable information, and promotes the trust between the nodes of the supply chain. Based on blockchain technology, Hou Defei et al. [9] designed a full life cycle traceability mechanism for aviation equipment quality and a federated chain network structure for aviation equipment quality management, constructed a full life cycle information chain of aviation equipment quality by using information technology data traceability descriptive model, and recorded the quality information of aviation equipment in various stages of design, production, transportation, maintenance, use, and end-of-life, which realized secure storage and information sharing of aviation equipment quality information. Feng Zhangwei et al. [10], in the research on new energy vehicle power battery traceability, adopted blockchain traceability technology to track the motivation of power battery usage and the influence mechanism of key factors, and the research found that the essential impact of blockchain technology on the closed-loop supply chain of new energy vehicles is positive. C. Shengfeng et al. [11], in order to solve the problems of low credibility and lack of information that exist in the current agricultural product traceability system due to centralized storage of data, found that blockchain technology has a significant impact on the closed-loop supply chain of new energy vehicles, positively affecting the closed-loop supply chain. The traceability information of agricultural products can be uploaded to the distributed ledger in real time, and the distributed storage of key traceability information can ensure the credibility and integrity of the traceability results. Zhang Jing et al. [12] integrated blockchain and IPFS technologies to design a multi-chain distributed traceability system to cope with the problems of the serious centralized structure of the traceability system of the agricultural supply market, fuzzy and rudimentary traceability information, and difficulty in effectively guaranteeing data security, combined with CBC and ECC encryption algorithms to provide encrypted protection of the privacy data needs, and improved the security and reliability of the off-chain storage of traceability information. H. Liu et al. [13] designed a dual-chain structure traceability system for rare Chinese herbal medicines by storing the detailed information of each process in the interstellar file system, storing the hash value of the traceability information of each production process in the private chain, and recording the transaction identifier ID in the private chain of the previous stage in the traceability information of the next stage so that the consumer can obtain the rare herbs with complete traceability information. S. Guo [14] constructed a traceability system for prepared vegetables based on blockchain technology in order to safeguard the quality and safety of prepared food products and enhance the effectiveness of transaction traceability, which ensured the safety and authenticity of the quality information of prepared food products and alleviated the thorny problem in the trade circulation of these products. Y. Sun et al. [15] proposed a blockchain-based traceability management for steel structure application scheme, which takes multiple parties involved in steel structure as nodes in the blockchain and sets up different data channels and permission levels for different business departments, and uses SM2 to encrypt and verify information and personnel, realizing efficient traceability and providing great assistance for steel structure projects. Y. Zhang et al. [16], in order to solve the problems of system centrality and the risk of malicious data tampering or destruction of mainstream product traceability platforms, proposed an application scheme based on blockchain, which can help to solve the problems of product traceability management. K. S. Rao et al. [17] addressed the centralization of the existing medical supply chain tracking and traceability system by proposing an effective Ethernet blockchain system for monitoring pharmaceutical products. blockchain system for monitoring medicines throughout the distribution chain to improve the traceability of the medicine supply chain. Liu Shannan et al. [18] proposed an organic rice supply chain traceability framework based on the federation chain and smart contracts to address the food safety issues and traceability accountability of organic rice and designed a data management model and smart contracts for each distribution link to realize the traceability and shareability of the organic rice supply chain.

Although the above research schemes solve the problem of centralization of the previous traceability system, they do not give security and integrity protection to the traceability information before it is stored on the chain, and the end result may affect the authenticity of the traceability results. In this paper, we propose a blockchain-based intellectual property transaction traceability scheme to cope with the problems faced by traditional intellectual property transaction traceability and propose solutions, which are mainly divided into the following two aspects:

Trace information asymmetry and opacity: In traditional IP transactions, buyers and sellers usually rely on intermediaries (e.g., lawyers, brokers, etc.) to conduct the transaction. This reliance may lead to information asymmetry, whereby the buyer is unable to fully understand key information such as the actual value, ownership, and usage history of the IPR. In addition, the opacity of the transaction process may make it difficult to identify the real owner of the IPR, its usage, or even whether there is a potential infringement issue. In this paper, we propose a scheme for collecting and describing the traceability information of intellectual property transactions based on PROV data origin model, which records the information of the whole life cycle of property rights through the origin model, integrates the traceability information of property rights, realizes the unity and transparency of the data format of the traceability information, solves the asymmetric and opaque problem of the traceability information among multiple parties of the transaction, and completes the automatic uploading of the traceability information by combining with the smart contract.

Authenticity and verifiability of traceability: Traditionally, the traceability of intellectual property rights mainly relies on paper documents, contracts, and government registration information, which makes it difficult to ensure the authenticity of the information. Falsification, alteration, or loss of records may make traceability difficult. Even with registration systems, such information is often insufficient or timely, especially in cross-border transactions, where records and legal systems in different countries and regions may also lead to traceability challenges. In the blockchain traceability system, the traceability data can be guaranteed to be tamper-proof on the chain, but the traceability information still faces the risk of being untrue before being uploaded to the chain. In this paper, we propose a BLS threshold signature-based verification scheme for traceability information of intellectual property transactions, which integrates the PROV model in the signature process, constructs a chain signature structure, and realizes credible on-chaining of traceability information of property rights transactions.

## 3. Blockchain-Based Intellectual Property Traceability Information Protection Scheme

### 3.1. General Structure

**Figure 1:**
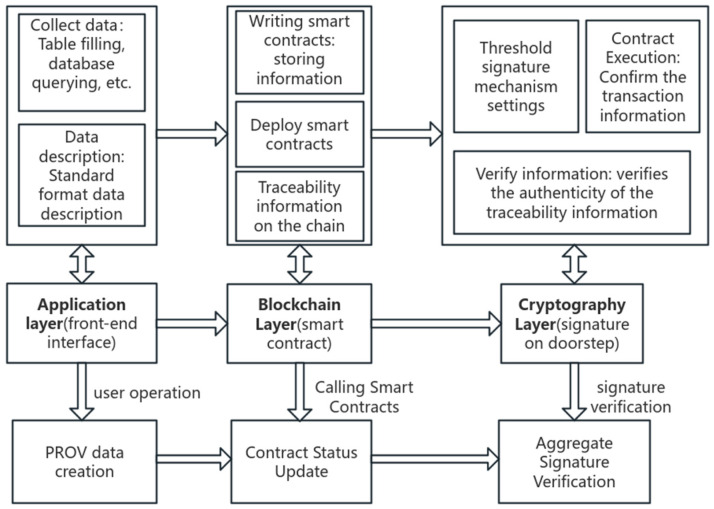
Overall architecture of information security mechanism for the traceability of intellectual property transactions.

Intellectual property transaction traceability needs to ensure the source is clear, transaction history, participant verification, and the transaction is not tamperable; for this reason, this paper proposes an intellectual property transaction traceability scheme based on blockchain and the PROV data model. The overall structure is shown in Figure 1. The flow of the scheme is divided into three stages: data collection stage, information encapsulation stage, and information verification stage. (1) The application layer completes the data collection and collects the record information of intellectual property rights in the three stages of property rights creation, property rights transaction, and property rights utilization through the PROV data model, which is the key to constituting the traceability information, and finally assembles into standardized intellectual property rights full life cycle traceability information after the data conversion through the PROV model. (2) The blockchain layer realizes the encapsulation of traceability information, i.e., the verified legal traceability information and legal signatures are stored on the chain through smart contracts, which are self-executing contracts whose terms and conditions are expressed in program code. This ensures the transparency and automated execution of transactions. Combining the PROV data model with smart contracts not only enhances the transparency and traceability of intellectual property transactions but also ensures the non-tampering of transaction records and automated execution. This approach can provide a more efficient and credible solution for the management and transaction of intellectual property rights. (3) In order to cope with the risk of tampering with traceability information before it is uploaded to the chain, a threshold signature scheme with an added time stamp is proposed to protect the uploaded traceability information securely. Considering the storage pressure of the blockchain system, the standardized traceability information file is stored on-chain and off-chain collaboratively, i.e., the file summary obtained from the hash calculation of the traceability information file is uploaded on the blockchain, and the traceability file containing a large amount of entity data is stored in the IPFS file system under the chain, the file address is further encrypted, and the ciphertext is uploaded on the chain. The traceability mechanism of the transaction is accomplished through the collaboration of smart contracts, blockchain networks, and off-chain databases, which users use to query traceability information. The user first applies a query request to the smart contract, queries the blockchain network through the traceability ID, gets the file address and file hash value of the traceability information, obtains the traceability information file in the IPFS database through the mapping relationship between the on-chain and off-chain, and then calculates the file hash, and then compares the result with the file summary on the chain to verify the verification results, which serves as a proof of the success of the query to complete the intellectual property rights. The verification result is used as proof of successful or unsuccessful query, finally completing the whole-life traceability of intellectual property transactions.

The program will be divided into three detailed steps: collection and description of traceability information for intellectual property transactions, encapsulation of traceability information into smart contracts, and verification of traceability information based on threshold signatures.

### 3.2. Collection and Description of Traceability Information of Intellectual Property Transactions

The PROV data model (provenance data model) is a standardized model for describing the origin of data (source, history, ownership and evolution process); the core goal is to achieve cross-system interoperability of data traceability information, combined with the data structure of PROV, the design of a specific signature format, embedding PROV information such as entities, activities, agents, etc. into the signature, making it possible to verify not only the validity of the signature but also the correct structure of the traceability information. Through a large number of intellectual property transaction process research and analysis, the common characteristics of intellectual property transaction information are mainly reflected in the following dimensions: (1) Subject and object. All of them involve basic attributes such as transaction parties (e.g., right holder, transferee, intermediary organization), intellectual property objects (patents, trademarks, copyrights, etc.), transaction time, transaction amount, right status (valid, invalid, disputed), legal jurisdiction, etc. It is also necessary to record the relationship between the subject of the transaction and the object (e.g., prov: was attributed to) and the history of changes in the right status (e.g., prov: was Disputed To, prov: was derived from). (2) Standardization of the transaction process. Most transactions need to go through standardized processes such as application, evaluation, contracting, registration, payment, delivery, etc. Some of them involve judicial or administrative filings. The timing relationship between process nodes needs to be recorded through prov: Activity (e.g., prov: was generated by indicates that the contracting activity generates the transfer of rights). (3) Legal compliance. All transactions need to comply with the legal framework of the Intellectual Property Law, Contract Law, etc., and the certification or approval behavior of the legal subject (e.g., court, intellectual property office) needs to be recorded through prov: Agent (e.g., Prov: acted On Behalf Of indicates the legality of the Agent). Through the PROV data model, a common and standardized traceability mechanism for intellectual property transactions can be constructed.

This stage is the basis for information tracing, mainly through the collection of data related to the various stages of intellectual property rights (creation, transaction, use).

1. Title creation stage:
  (1) Record the creator’s identifying information, time of creation, and place of creation;
  (2) Confirmation of the act of creation by means of electronic signatures, etc., and generation of related metadata.
  (3) Utilizing the PROV model, in which prov: Entity represents the created work, prov: Activity represents the creation behavior, and prov: Agent represents the creator.
2. The stage of property rights transactions:
  (1) Record information on transactions such as the purchase, sale, authorization, and licensing of intellectual property.
  (2) Include information such as the time of the transaction, the counterparty, and the price of the transaction.
  (3) In PROV, prov: Entity denotes the intellectual property of the transaction, prov: Activity denotes the transaction behavior, and prov: Agent denotes the counterparty.
3. Property use stage:
  (1) Record the actual use of intellectual property rights, including the party that uses it, when it is used, and how it is used.
  (2) In PROV, prov: Entity denotes the intellectual property being used, prov: Activity denotes the usage behavior, and prov: Agent denotes the user.

In this process, various types of information are collected in real time through smart sensors, contracts, or other means to ensure the authenticity and validity of the data.

The whole life cycle of intellectual property rights, from the creation of property rights to the trading of property rights to the use of property rights, involves multiple participants, and the data types of the information involved are not uniform but closely related. For example, intellectual property rights may also have format changes during the transaction process, and the data types of traceability records submitted by each participant are inconsistent, resulting in data heterogeneity during the traceability process of the whole life cycle of intellectual property rights. In order to better adapt to the transaction and traceability of intellectual property rights, ensure the comprehensiveness of intellectual property rights traceability information, and improve the efficiency of property rights transaction traceability, a general and reliable data traceability model is needed. In this paper, based on the consideration of intellectual property transaction scenarios, the PROV data model is adopted to collect and process information on the full life cycle traceability of intellectual property.

By analyzing the participating subjects in the three stages of creation, transaction, and use of intellectual property rights as well as the activities of the transaction process, the corresponding description of the information of the intellectual property transaction process in the PROV model is designed, as shown in Table 1.

### 3.3. Traceability Information Encapsulation Smart Contract

Smart contracts are self-executing contracts whose terms are represented by program code that ensures transparency and automated execution of transactions. Combined with the PROV data model, smart contracts can be used to store and manage traceability information for IP transactions.

It is first necessary to define the data structure in the smart contract to store the entity, activity, and participant information in the PROV data origin model. The design of the smart contract part of the traceability information of the intellectual property transaction is represented by the Solidity structure, which is created to record the traceability information of the intellectual property. This specifically includes the entity, activity, agent, and association between the entity and the activity:

In the PROV data model, an entity represents a data object or resource, which can be any intellectual property rights that need to be traced, such as patents, trademarks, copyrights, and so on. In the smart contract, a structure needs to be defined for each IPR entity, containing the basic information of the entity. Id represents the unique identifier of the entity, which is used to differentiate different entities; name represents the name of the entity, which is usually the name of the IPR; and description represents the detailed description of the entity, which is capable of describing the specific content of the IPR.

An activity represents the process of creating, modifying, transferring, etc., data or entities. In the scenario of intellectual property rights, the activity can involve the application, license, transfer of patents, and so on. In the smart contract, a structure is defined for each activity, which contains detailed information about the activity. It represents the unique identifier of the activity, which distinguishes different activities; typeOfActivity represents the type of the activity, which can be “create”, “transfer”, “license”, etc. This field indicates how the activity affects the entity; timestamp indicates the timestamp of the occurrence of the activity, recording the specific time at which the activity took place; agent indicates the agent that performs the activity, usually the participant that performs the activity (e.g., owner, buyer, seller of intellectual property, etc.).

In the PROV model, an agent denotes a subject involved in an activity, such as an individual, organization, or system that performs the activity. In a smart contract, an agent can be an address, usually the wallet address of the person performing the activity. Id denotes the unique identifier of the agent, which is used to distinguish between different agents; agent address denotes the address of the agent, usually an Ether address, which denotes a participant in the execution of the activity. Role denotes the role of the agent, which can be “creator”, “buyer”, “seller”, etc., which defines the agent’s specific responsibilities in the transaction.

Activities usually affect or generate entities. Therefore, a mechanism is defined in a smart contract to associate entities with activities. In a smart contract, this can be accomplished through mapping. entityActivities refers to mapping relationships that store the IDs of an entity and its associated activities. Each entity (uniquely identified by Entity.id) may be associated with multiple activities (uniquely identified by Activity.id).

The functionality of the smart contract is strong and expandable, and the corresponding functional design can be made according to specific needs. The following describes the functional design of the smart contract for intellectual property transaction traceability information:

Create Entity: Use the createEntity function to create a new entity (e.g., patent) and record its basic information.

Create Activity: Use the create activity function to create an activity related to an IP transaction (e.g., assignment, license, etc.).

Create Agent: Use the createAgent function to record information about the agents involved in the activity (e.g., creators, buyers, etc.).

Associate Activity with Entity: Associate an activity with an entity through the associateActivityWithEntity function, which records the activity that occurred at a specific time for that entity.

Get Entity Activities: Using the getEntityActivities function, you can query all the activities related to a certain entity, supporting traceability queries.

Finally, blockchain technology is utilized to verify the collected information. The smart contract will verify the authenticity of the traceability information based on preset rules and generate a unique identifier (e.g., a hash value) as a signature for the information.

**Figure 2:**
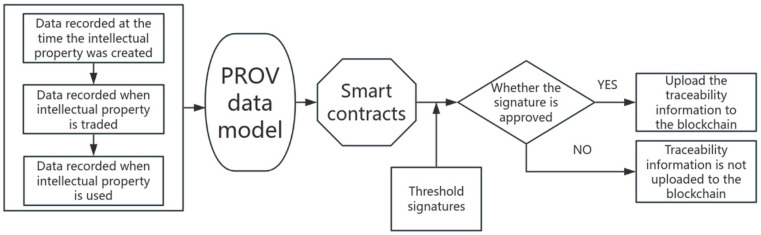
Intellectual property rights traceability information flow path.

Through the PROV data model after processing the various stages of record information into a unified description of the traceability information, the conversion of data relies on the realization of the smart contract with the contract encapsulates the intellectual property rights of all participants in the exchange for the read and write logic of the traceability information on the blockchain chain, and then the smart contract will be deployed on the blockchain, the installation of all the nodes in the channel. Figure 2 below shows the flow path of traceability information and, finally, through the signature verification, determines whether the traceability information is on the chain or not.

The standardized traceability information obtained through the PROV data model needs to be further verified by the threshold signature scheme before it can be uploaded to the blockchain, and the smart contract will judge the uploading status of the traceability information according to whether the signature result passes or fails, i.e., if the signature succeeds, then the traceability information will be uploaded to the blockchain; if the signature fails, then the traceability information cannot be uploaded. The main role of the smart contract in the uploading stage of traceability information is to complete the unified conversion of data recorded in the creation, transaction, and use stages of intellectual property rights into a standardized JSON format and upload the traceability information after the threshold signature is successful.

**Figure 3:**
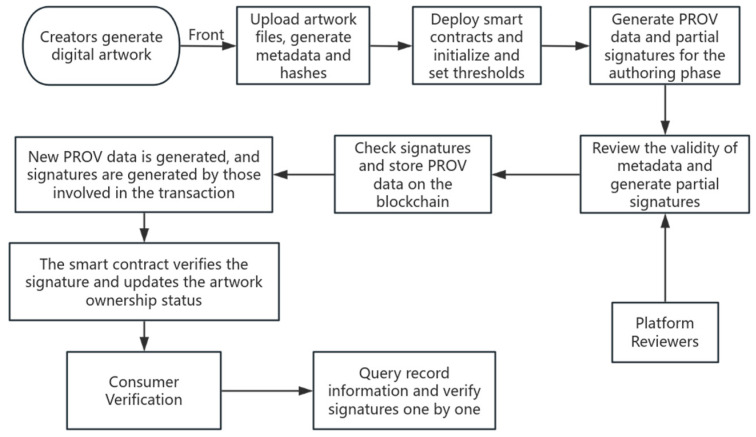
Flow of simulation scenario for digital artwork traceability.

Figure 3 below analyzes the application of the PROV model in the traceability process by simulating the digital artwork traceability scenario. First, the creator of the digital artwork uploads the original data of the digital artwork (pictures, videos, etc.) through the front-end interface, generates the metadata and hash value, and the creator sets the threshold signature threshold value through the smart contract. The metadata of the digital artwork generates the traceability data of the artwork at the creation stage through the prov data model, and after the trading platform audits the legitimacy of the traceability information (multiparty signature authentication), it submits the traceability information of the creation stage (the creation time of the digital artwork, the identity of the creator, the metadata of the artwork, and other key information) and some of the signatures to the blockchain for storage. After the artwork is traded, new PROV data (including timestamps and partial signatures of the previous stage) and partial signatures are generated, the legitimacy of the signatures is verified through smart contracts, and the ownership of the digital artwork is updated at the same time. The use of the digital artwork (e.g., secondary creation or commercial use) is still recorded through PROV, and the smart contract is invoked to upload the blockchain after the signature is passed. Consumers or other verifiers can verify the signature through PROV data information and realize the credible query of traceability information.

### 3.4. Authentication of Traceability Information Based on Threshold Signatures and Timestamps

The signature verification of the trace information is completed based on the BLS threshold signature. On this basis, the BLS is extended to the threshold scenario, and the security is generalized to the CDH and the random predicate machine, and Boldyreva, A. et al. [19] have made the security proof. Combining the intellectual property transaction scenario, assigning signature membership identifiers, signature integration with the PROV model, and constructing a chained signature structure avoid single point of failure through decentralized key management; the PROV structure is deeply bound to the signature to ensure the integrity of the data in each link; the bilinear pair characteristic is used to achieve fast batch verification and improve the credibility and efficiency of the signature. The signature process is divided into four steps: system initialization, distributed key generation, threshold signature generation, and signature verification.

#### 3.4.1. System Initialization

(1) Define parameters

Bilinear group: choose G1, G2, GT as the cyclic groups of prime order *p*. Generate the element g ∈ G1, ℎ ∈ G2, the bilinear mapping: G1 × G2→GT; hash function: H:{0,1}*→G1, the message mapping with G1.

(2) Role identification

Number of signers n = 5, Threshold t = 3, i.e., set five signers for verification of intellectual property traceability information, as shown in Table 2.

#### 3.4.2. Distributed Key Generation

The five participants involved in the signature jointly generate the private key to avoid centralization:

Each signing participant i selects the polynomial: $f_{i}(x)={a_{0}}^{\left(i\right)}+{a_{1}}^{\left(i\right)}x+\cdots +{a_{t-1}}^{\left(i\right)}x^{t-1}$.

Broadcast Commitment ${\left\{h^{{a_{k}}^{\left(i\right)}}\right\}}_{k=0}^{t-1}\in G_{2}$.

Send a secret share to the signature participant $js_{i,j}=f_{i}(j)modp$.

Verify share: check $h^{s_{i,j}}=\prod_{k=0}^{t-1}{\left(h^{{a_{k}}^{\left(i\right)}}\right)}^{j^{k}}$.

Each member $P_{i}$ computes its own private key slice:

$${sk}_{i}=\sum_{j=1}^{n}s_{j,i}modp$$

The system’s public key is as follows:

$$pk=h^{\sum_{i=1}^{n}{a_{0}}^{\left(i\right)}}=\prod_{i=1}^{n}h^{{a_{0}}^{\left(i\right)}}\in G_{2}$$

#### 3.4.3. Threshold Signature Generation

(1) PROV message construction

The traceability information for each process (title creation, title transaction, title use) is encoded as a PROV triad (IP is mapped to the elements of the PROV model, i.e., entity, activity, agency), and the traceability information is structured:

$$m_{process}=PROV\_Entity∥PROV\_Activity∥PROV\_Agent∥Timestamp$$

Hash processing: $H(m_{process})\in G_{1}$.

(2) Partial signature generation

Participant i generates a partial signature to the traceability message m:

$$\sigma_{i}={H\left(m\right)}^{{sk}_{i}}\in G_{1}$$

(3) Signature aggregation

By the definition of the Lagrange interpolation formula, at a given t points $\left(x_{1},y_{1}\right)$, $\left(x_{2},y_{2}\right)$, …, $\left(x_{t},y_{t}\right)$, where $y_{i}=f(x_{i})$, the value of the polynomial f(x) at the point x=0, can be recovered by the following formula:

$$f(0)=\sum_{i=1}^{t}y_{i}\cdot {\lambda_{i}}^{S,0}modp$$

f(0) is positively represented here as the full private key sk. Thus, after collecting at least three partial signatures, the full signature can be recovered using Lagrange interpolation:

$$\sigma =\prod_{i\in S}{\sigma_{i}}^{{\lambda_{i}}^{S,0}}\in G_{1}$$

$$\sigma =\prod_{i\in S}{\left({H\left(m\right)}^{{sk}_{i}}\right)}^{{\lambda_{i}}^{S,0}}={H\left(m\right)}^{\sum_{i\in S}{\lambda_{i}}^{S,0}{sk}_{i}}={H\left(m\right)}^{sk}$$

where ${\lambda_{i}}^{S,0}$ denotes the Lagrangian coefficient:

$${\lambda_{i}}^{S,0}=\prod_{\begin{array}{c} j\in S\\ j\neq i \end{array}}\frac{0-j}{i-j}modp$$

#### 3.4.4. Signature Verification

To ensure the continuity of creation, transaction, and use, each stage signature contains the hash of the previous stage signature, forming a chained signature structure, i.e.,

Intellectual Property Creation Stage Signature ($\sigma_{create}$) Signature $m_{create}$;

IP Transaction Stage Signature ($\sigma_{transfer}$) Signature $m_{transfer}∥H\left(\sigma_{create}\right)$;

Intellectual Property Use Stage Signature ($\sigma_{use}$) Signature $m_{use}∥H\left(\sigma_{transfer}\right)$.

Verify that the aggregated signature σ was generated by a legitimate participant and has not been tampered with.

Calculate the message hash and hash the message m to the group $G_{1}$:

$$h_{m}=H\left(m\right)\in G_{1}$$

Verification of bilinear pairwise equations:

$$e\left(\sigma ,h\right)=e\left(H\left(m\right),pk\right)$$

According to the aggregated signature formula,

$$\sigma ={h_{m}}^{\sum_{i\in S}{\lambda_{i}}^{S,0}{sk}_{i}}$$

Bilinear to the left:

$$e\left(\sigma ,h\right)=e\left({h_{m}}^{\sum_{i\in S}{\lambda_{i}sk}_{i}},h\right)={e\left(h_{m},h\right)}^{\sum_{i\in S}{\lambda_{i}sk}_{i}}$$

Bilinear to the right:

$$e\left(h_{m},pk\right)=e\left(h_{m},h^{\sum_{i\in S}{a_{0}}^{\left(i\right)}}\right)={e\left(h_{m},h\right)}^{\sum_{i\in S}{a_{0}}^{\left(i\right)}}$$

As

$$\sum_{i\in S}{\lambda_{i}sk}_{i}=\sum_{i\in S}{a_{0}}^{\left(i\right)}$$

It can be verified that the equation holds, and if the equation holds, it passes the validation, thus ensuring that the IP transaction traceability information has not been tampered with.

#### 3.4.5. Threat Model Assumptions and Defense Strategies

1. Witch Attack: The attacker forges multiple false identities (nodes) to participate in the system in an attempt to control the network or the signature process.

Defensive strategies: (1) Staking economic model (Staking), the mechanism is that each authentication node needs to pledge assets S (such as tokens), and malicious behavior will lead to forfeiture (Slashing). Assuming that the cost for an attacker to create x fake nodes is C=x·S, and if the attack is detected, the forfeiture amount is set to P·C, then the expected loss of the attack is as follows:

E[loss]=P·Pr−C

Pr is a detectable risk when P·Pr>C, the attack is unprofitable.

(2) Decentralized Identity Binding (DID), where nodes bind off-chain identities through decentralized identifiers, and the uniqueness of identities is guaranteed through hash commitments:

$$Commit=H(DID∥Nonce)\in G_{1}$$

DID (user ID or personal public key) is bound to Nonce (random number), and duplicate identities will lead to hash conflicts and anti-guaranteed uniqueness for each calculation.

2. Data forgery: Attackers tamper with PROV data, such as artwork metadata and transaction records.

Defense strategy: (1) Chained hash binding, where the hash value of each data block $D_{i}$ is embedded in the next stage signature.

The signature is generated with a preordered hash included:

$$\sigma_{i}={H\left(D_{i}∥H\left(D_{i-1}\right)\right)}^{{sk}_{i}}\in G_{1}$$

Verify the equation:

$$e\left(\sigma_{i},h\right)=e\left(H\left(D_{i}∥H\left(D_{i-1}\right)\right),pk\right)$$

When the equation holds, it indicates that the current data is authentic; otherwise, it indicates that the data has been tampered with and has lost its legitimacy.

(2) Add timestamps to avoid replay attacks. The timestamp identifier is embedded in the hash calculation of the message:

$$H(D)=H\left(date∥Timestamp\right)$$

3. Collusion attack: Some nodes collude in an attempt to forge signatures or tamper with data.

Defense strategy: (1) The threshold protocol strictly requires at least t valid signature fragments to generate a legitimate signature (described in Signature Aggregation above).

(2) Dynamic node rotation: Periodically, some nodes are replaced by DAO voting to prevent long-term collusion. The probability that a node is replaced during the rotation period T is as follows:

$$Pr(replace)=1-{\left(1-\frac{k}{n}\right)}^{T}$$

where k is denoted as the number of substitutions per cycle.

4. Smart contract vulnerability: Unauthorized operations using contract code vulnerabilities.

Defensive strategies: (1) Formal validation, using the tool Certora to transform the contract code into a mathematical model to verify compliance with security properties.

(2) Monitor abnormal behavior on the chain (e.g., abnormal transfer of large sums of money, abnormal invocation of contracts) and track transaction data through the blockchain browser.

## 4. Experimental Analysis

### 4.1. Performance Analysis

This article delineates a protection program for the traceability of intellectual property, the formal proof scheme satisfies both unforgeability and forward security, and a new threshold signature aggregation formula is proposed to optimize the efficiency of signature recovery through the Lagrangian interpolation coefficient. Intellectual property traceability is divided into three stages: creation, transaction, and use. The traceability information structure is constructed through the PROV data model, the signer’s identity is assigned, and the hash value of the preceding stage is embedded in the signature to form a tamper-evident chained signature structure, which ensures the continuity and traceability of intellectual property rights and defends against man-in-the-middle attacks.

**Figure 4:**
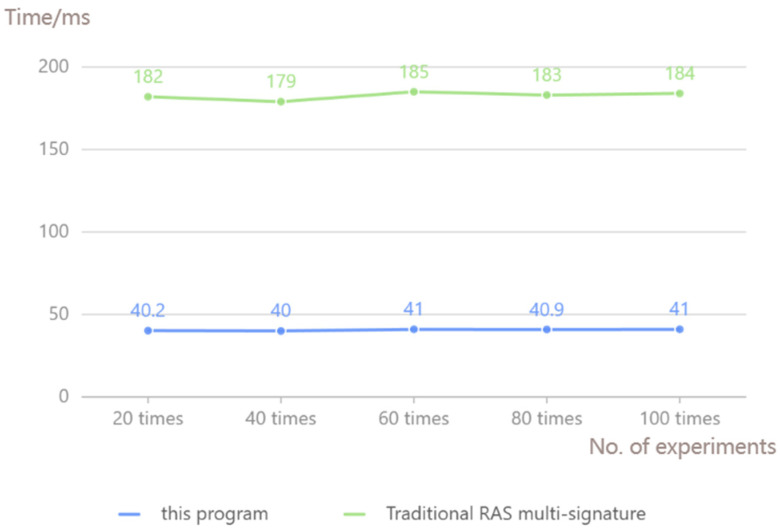
Signature aggregation time comparison.

In the context of a multiparty signature, the signature aggregation scheme and the traditional RSA multi-signature comparison are in the same network environment under the conditions of 100 tests. Figure 4 shows the signature aggregation time comparison. It can be seen that the range of signature aggregation time of this scheme is 37~43 ms, with an average time of 40 ms, and the average time of signature aggregation of traditional RSA multi-signature scheme is about 180 ms, and the efficiency of partial signature aggregation of this scheme is more superior in the case of a single part of the signature completion time is the same.

**Figure 5:**
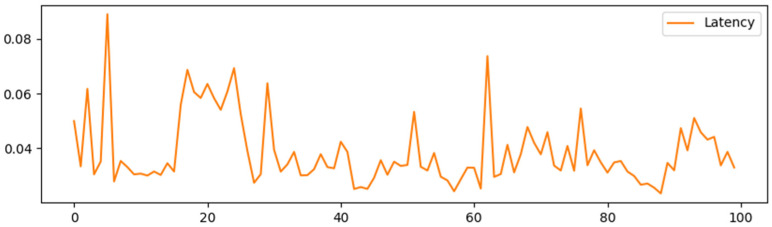
Delay time for 100 single message validations.

**Figure 6:**
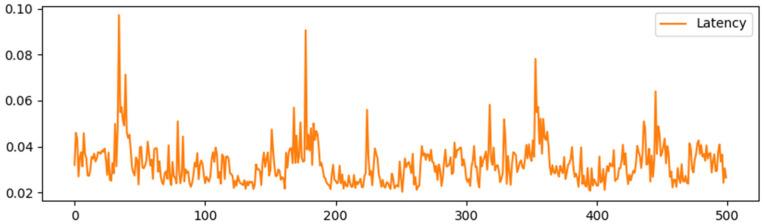
Delay time for 500 single message validations.

**Figure 7:**
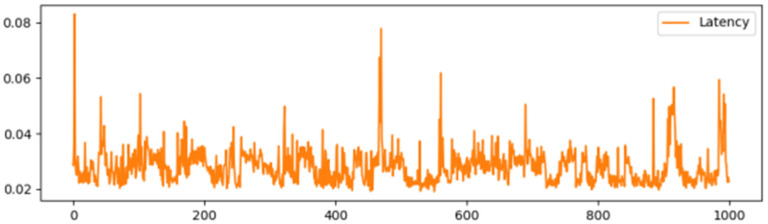
Delay time for 1000 single message validations.

**Figure 8:**
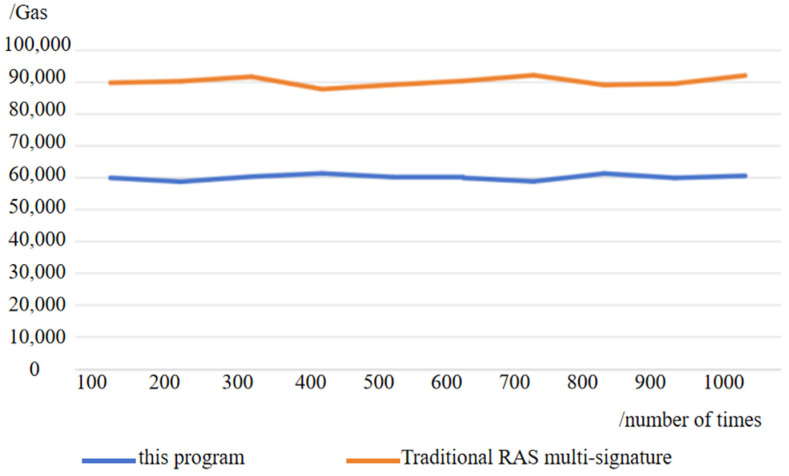
Comparison chart of single verification gas consumption.

Simulating the Ethernet network through the Ganache local test, Figure 5, Figure 6, Figure 7 and Figure 8 show the delay time and Gas consumption of a single transaction completed by the test transaction verification under the test condition of concurrent transactions increasing from 100 to 1000, respectively. The data shows that the average delay of single transaction information verification is around 35 ms, and the value of Gas is around 6000 on average. Comparing the average delay of 60 ms and gas consumption of around 9000 under the traditional RSA multi-signature condition shows the superiority of this scheme in terms of performance.

### 4.2. Comparison of Traceability Information Protection Programs

In this paper, we compare the traceability information security schemes among other traceability schemes in terms of data relevance, dynamic extensibility, and security, and the results are shown in Table 3.

Compared with the aquatic products trading traceability data system model proposed in the Ref. [7], the intellectual property chained traceability information structure proposed in this paper has good traceability continuity, which can cope with the requirements of the integrity of trading traceability information in different contexts and improve the reliability of the traceability system.

Compared with the traceability schemes proposed in Refs. [9,12], although the standardized model of traceability data is realized, the degree of security protection of its traceability information is not enough. Although Refs. [20,21] constructed a hierarchical traceability information storage and query model, the correlation of whole-life traceability information is weak, which makes it easy for malicious tampering of intermediate traceability information to occur. The threshold signature protection scheme proposed in this paper, with the deep coupling of the PROV data model and BLS signature, solves the problem of data silo and single-point trust in the traditional scheme.

In addition, this paper’s scheme still has unique advantages when compared to the 5G version of Distributed Blockchain-based Trusted Multi-Domain Collaboration for Mobile Edge Computing, Blockchain-based Three-Party Anonymization Identification for Trusted Services in the Industrial IoT, Blockchain-based Secure Distributed Control in Software-Defined Optical Networks, and the Blockchain-based Jointcloud Layered Trust Network.

Distributed Blockchain-based Trusted Multi-Domain Collaboration utilizes blockchain to achieve cross-domain (e.g., operator, edge node, user device) resource scheduling and trust management, combined with zero-knowledge proof for privacy protection. This paper’s scheme realizes finer-grained traceability in cross-domain collaboration through BLS threshold signatures and PROV chained structure, while existing 5G schemes mostly rely on simple hash chains, which are unable to trace the timing of multiparty operations. Meanwhile, this scheme has better latency than traditional RAS multiparty signatures, which is suitable for edge computing with low latency requirements.

Blockchain-based three-party anonymous identification in industrial IoT realizes three-party anonymous interactions among devices, gateways, and cloud platforms through ring signatures or group signatures to ensure privacy and auditability. This scheme accurately tracks abnormal device operations through the input-output chained hash structure, which is superior to the coarse-grained auditing of group signatures.

Blockchain-based secure distributed control in software-defined optical networks utilizes smart contracts to achieve dynamic allocation of optical network resources (wavelengths, routes) to prevent DDoS attacks. The scheme in this paper ensures the legitimacy of control commands (e.g., wavelength allocation requires 3/5 administrators’ signatures) through threshold signature verification, while existing schemes mostly rely on a single controller, which poses a risk of single-point attacks.

Blockchain-based Jointcloud layered trust network constructs a layered trust architecture across cloud service providers and realizes resource scheduling and billing auditing through blockchain. The PROV model in this paper supports multi-layer traceability of cross-cloud operations, while the existing scheme in Jointcloud only records the resource allocation results and lacks process tracking. Moreover, this scheme quickly locates the failure level through chained hashing, and the auditing time is reduced from hours to minutes. In terms of billing transparency, each service call generates a PROV record and signature to prevent billing fraud.

### 4.3. Experimental Tests

The experimental test environment is off-chain: Ubuntu 20.04.3 LTS, Node.js v18.20.6, on-chain: Solidity ^0.8.0, Remix IDE v.0.64.0 (JavaScript VM environment).

**Figure 9:**
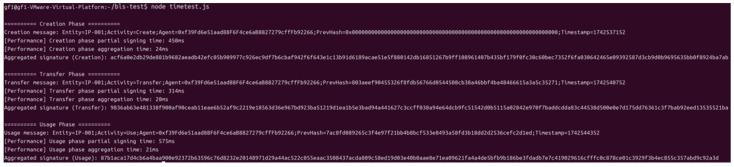
Intellectual property traceability information defined by the prov model.

The test script traces intellectual property through three stages: creation (create), transaction (transfer), and use (use), generates partial signatures at each stage, then aggregates these signatures, measures the time to generate signatures and the time to aggregate at each stage, and outputs them to the console. Figure 9 shows the three stages of traceability information (create, transfer, and use), traceability information structure (entity, activity, and agent), signature, and private key information.

**Figure 10:**
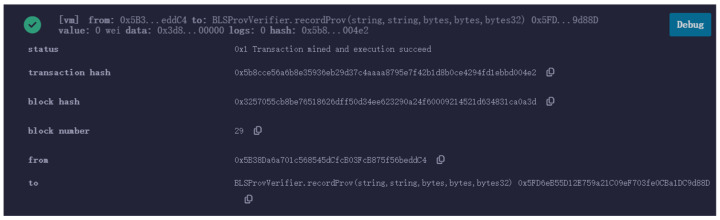
Property rights traceability information stored in the blockchain.

Deploy the smart contract in Remix IDE and enter the recordProv information to submit this transaction request. Figure 10 below shows the submitted and packaged transaction information.

**Figure 11:**
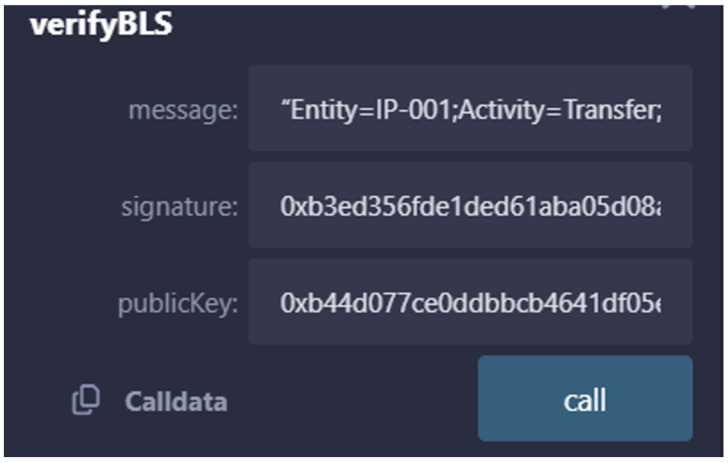
Information required for signature verification.

The result of the transaction message signature is verified by the verifyBLS verification function, and Figure 11 shows the parameters required for verification.

**Figure 12:**
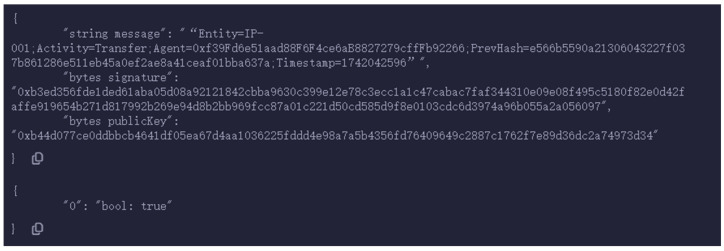
Signature verification passed.

Figure 12 demonstrates that the verification result is true, which means that the signature verification has passed and the information has not been tampered with.

## 5. Conclusions

In this paper, for the problems of opaque intellectual property transaction traceability and easy tampering of traceability information, a chain traceability scheme integrating the BLS threshold signature and the PROV model is proposed. Experiments show that the efficiency of signature aggregation in the same scenario is significantly improved compared with RSA multi-signature. The chain construction of traceability information in PROV provides a data integrity guarantee for intellectual property transaction traceability. The seamless combination of the BLS threshold signature and the PROV model guarantees the security and trustworthiness of intellectual property’s full life cycle traceability. However, this paper has bottlenecks in computation, communication, and storage, the computational overhead of signature verification, the high handling fee and storage pressure caused by transaction data expansion, etc., and the second-layer blockchain solution will be the direction of continued efforts.

## Figures and Tables

**Table 1:** Correspondence of intellectual property transaction information in the PROV data model.

Intellectual Property Transaction Information	PROV Data Model Elements	Corresponding Description
Intellectual property entities (e.g., patents, copyrights, etc.)	Entity	Intellectual property rights themselves are represented in the PROV model as entities. For example, information such as the number, description, time of creation, and holder of a patent can be considered as entities.
IP creators (creators, inventors) and IP holders (e.g., companies or individuals)	Agent	The creator or inventor of intellectual property is the agent who performs the creation activity and participates in the process of generating intellectual property as the relevant agent. The holder of the intellectual property rights acts as an agent in the transaction and may be an individual, a business, or another organization.
Creation, trade, and use	Activity	Describes the flow of activities of IP entities in different scenarios, including the creation of property rights, the trading of property rights, and the use of property rights.
Parties to the transaction (e.g., buyer, seller, agent)	Agent	In an IP transaction, buyers, sellers, agents, etc. can act as agents, executing the transaction activities and associating with the corresponding IP entities.

**Table 2:** Role responsibilities and trust assumptions.

Role	Duty	Trust Assumptions
Creator	Generate digital artwork metadata to initiate the initial signature process.	Assume a trusted entity, but use authentication (such as a digital certificate) to prevent identity forgery.
Validators	Participate in distributed key generation (DKG); validate and sign transactions or usage records.	The nodes may be partially Byzantine (malicious), but the total number does not exceed the threshold t − 1
Platform Auditor	Review the legitimacy of metadata (e.g., copyright ownership, content compliance) and participate in multi-party signatures.	It needs to be elected through an on-chain governance mechanism, assuming that its behavior is subject to economic staking (such as staking).
Buyers or consumers	Buy or use digital artwork, verify historical signatures, and initiate transaction signatures.	It is possible that a rational attacker (attempting to tamper with ownership) relies on cryptography to constrain its behavior.
Auditors	Independently verify PROV chain integrity and detect anomalous behavior.	There is no need to hold a key, only need to read the on-chain data, assuming that it has compliance review capabilities.

**Table 3:** Comparison of traceability information models.

Programmatic	Data Relevance	Dynamic Scalability	Safety
[7]	weak	medium	weak
[9]	Strong	medium	medium
[12]	medium	weak	medium
[20]	medium	Strong	weak
[21]	weak	medium	medium
this program	Strong	Strong	Strong

## Data Availability

The data used to support the findings of this study are included within the article.
